# Respiratory viral infections and host responses; insights from genomics

**DOI:** 10.1186/s12931-016-0474-9

**Published:** 2016-11-21

**Authors:** Niamh M. Troy, Anthony Bosco

**Affiliations:** Telethon Kids Institute, The University of Western Australia, Subiaco, Australia

**Keywords:** Respiratory viral infections, Host response, Genomics, Respiratory syncytial virus, Rhinovirus, Influenza, Gene network, Systems biology

## Abstract

Respiratory viral infections are a leading cause of disease and mortality. The severity of these illnesses can vary markedly from mild or asymptomatic upper airway infections to severe wheezing, bronchiolitis or pneumonia. In this article, we review the viral sensing pathways and organizing principles that govern the innate immune response to infection. Then, we reconstruct the molecular networks that differentiate symptomatic from asymptomatic respiratory viral infections, and identify the underlying molecular drivers of these networks. Finally, we discuss unique aspects of the biology and pathogenesis of infections with respiratory syncytial virus, rhinovirus and influenza, drawing on insights from genomics.

## Background

Respiratory infections are the leading cause of disease globally [1]. In young children, they are responsible for around one quarter of hospitalizations and up to 60% of visits to the general practitioner [2]. In most cases, respiratory viral infections are self-limiting and confined to the upper airways, where they evoke relatively mild symptoms such as sneezing and a runny nose [3]. However, in susceptible individuals, such as newborns and the elderly, the symptoms can impact on the lower airways, resulting in wheeze, shortness of breath, bronchiolitis or pneumonia [4]. Common respiratory viral pathogens include adenovirus, enterovirus, human coronavirus, human metapneumovirus, rhinovirus (RV), influenza, parainfluenza and respiratory syncytial virus (RSV). Of these viruses, most genomic studies have focused on RSV, RV and influenza. This is because RSV is the most important cause of respiratory tract infections leading to hospitalization among infants [5]. RV causes the majority of colds and around two thirds of asthma exacerbations [6]. Influenza is the most common cause of pneumonia related deaths in developed countries [7]. The mechanisms that determine why some individuals suffer from severe illness whilst others do not are not well understood. In this review we will focus primarily on the contribution of the host response, drawing on insights from genomics.

### Gene expression profiling technologies

The advent of microarrays enabled the measurement of gene expression patterns on a genome-wide scale [8]. Microarrays comprise a vast array of oligonucleotide probes of 25 bases or more in length, fixed to a solid surface. The probes bind to labelled target molecules (e.g. cDNA derived from mRNA), and the signal intensity is quantified. Despite this major technological advance, cross-hybridization of related sequences limited the signal-to-noise ratio [9]. Microarrays are slowly being phased out and superseded by deep sequencing technologies for gene expression profiling (RNA-Seq). RNA-Seq entails the generation of a library of cDNA fragments from total RNA or mRNA, followed by ligation of adapters, PCR amplification and high throughput sequencing [10]. This generates millions of short sequencing reads (e.g. 50–200 bases), which are aligned to a reference genome sequence, and summarized as gene- or transcript-level counts. Compared with microarrays, advantages of RNA-Seq include single base resolution, superior sensitivity and dynamic range, and the ability to detect novel transcripts. The data from microarray and RNA-Seq experiments is typically submitted to a public repository at the time of publication (e.g. Gene Expression Omnibus; http://www.ncbi.nlm.nih.gov/geo/; Sequence Read Archive; www.ncbi.nlm.nih.gov/sra), allowing other researchers to freely access the data for reanalyses [11].

### Network analysis of genomic data

Most genomic studies focus on identification of differentially expressed genes. However, this approach is limited, because genes do not exist nor function in isolation, they work together [12]. A significant advance in this context was the application of network graph theory to genomic data analysis [13]. The underlying concept is that a functioning biological system can be represented as a network of interconnected nodes and links. The nodes represent genes or their products (mRNA transcripts, proteins) and the links represent functional or statistical relationships between genes. For instance, the links could represent physical binding interactions between proteins, or coexpression relationships between mRNA transcripts. Gene networks have a non-uniform, so-called “scale-free” topology, where most genes are connected to few genes, and a few genes are connected to many [14]. This results in a limited number of highly connected nodes called hubs, which essentially “hold” the network together [14]. An emergent property of scale-free networks is a tolerance to random perturbations and a susceptibility to the disruption of hubs [13]. Another fundamental organizing principle of gene networks is modularity, meaning that genes which function in the same biological process form densely interconnected subgraphs embedded within the network structure [15]. Modules execute biological tasks, and the breakdown of functional modules is thought to underpin disease states [16].

### Respiratory viral pathogens and entry mechanisms

RSV is an enveloped, single strand, negative sense RNA virus of the family *Paramyxoviridae*. There are two antigenic subtypes (RSV-A, RSV-B). The genome encodes seven structural proteins and four non-structural proteins. The envelope is encoded by three glycoproteins, namely the small hydrophobic protein, the attachment protein G and the fusion protein F. Virus entry is mediated by binding of the F protein to host-cell nucleolin [17].

RV is a non-enveloped, single strand, positive sense RNA virus belonging to the *Picornaviridae* family. It is contained within an icosahedral capsid, encoded by four viral proteins (VP1-VP4). More than 160 strains have been identified, and these are classified into three species (RV-A, −B, −C) on the basis of genome sequence. RV-B and most RV-A strains bind intercellular adhesion molecule (ICAM1) on the cell surface to gain entry. A subgroup of RV-A strains bind to low-density lipoprotein receptor (LDLR) family members for virus entry [18]. The entry mechanism for RV-C remained elusive until recently. Using microarrays, it was demonstrated that expression of cadherin-related family member 3 (CDHR3) was upregulated in cells that were susceptible to RV-C versus cells that were resistant [19]. Moreover, ectopic expression of CDHR3 in resistant cells conferred susceptibility to infection.

Influenza is an enveloped virus from the *Orthomyoxoviridae* family. The genome is composed of eight segments of single strand, negative sense RNA. It is classified into three subtypes (A, B, C). Influenza A virus (IAV) is the major subtype that circulates in humans, but it can also infect a diverse range of hosts including mammals and birds. The IAV genome encodes 11 proteins; hemagglutinin (HA), neuraminidase (NA), nucleoprotein (NP), matrix proteins (M1, M2), non-structural proteins (NS1, NS2), polymerase proteins (PB1, PB2, PA), and PB1-F2 and is further classified based on the HA and NA glycoproteins found on the viral envelope. Glycans containing sialic acid mediate IAV attachment. Seasonal IAV binds to ASα2-6Gal, which are found primarily on the surface of epithelial cells in the nasal mucosa, trachea and bronchi. In contrast, highly pathogenic avian H5N1 virus binds to ASα2-3Gal, which is found on alveolar epithelial cells [20].

### Innate immune sensing of viral infections

**Fig. 1:**
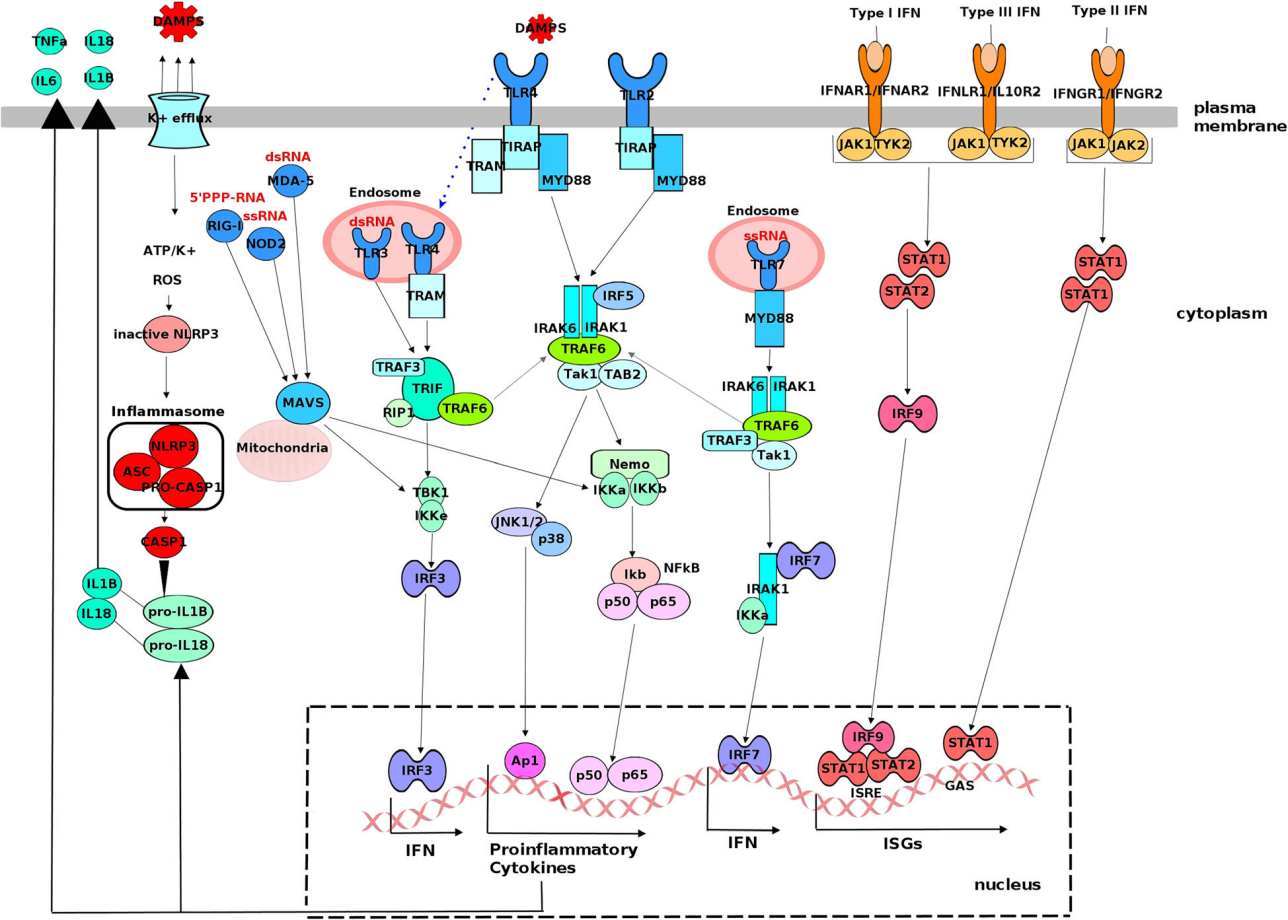
Viral sensing pathways that trigger host immune responses. PRR signalling is triggered by viral proteins and nucleic acids (PAMPs) and host-derived DAMPs. All TLRs except TLR3 signal through the adapter molecule Myd88. TLR3 signals through TRIF, and TLR4 signals via both Myd88- and TRIF-dependant pathways. RIGI, MDA5 and NOD2 signal through the mitochondrial antiviral-signaling protein MAVS (or IPS1). These signalling pathways converge on IRF3/IRF7 and NFkB to activate their respective type I/III interferons and proinflammatory gene programs. Type I (IFN-α, IFN-β) and III interferons (IFN-λ1, IFN-λ2, IFN-λ3 or IL-29, IL-28A, IL-28B) bind to distinct receptor complexes, which activates STAT1 and STAT2 phosphorylation. IRF9 binds to STAT1/STAT2 heterodimers forming the ISGF3 complex, which translocates to the nucleus to induce transcription of ISGs that contain ISRE elements in their promoters. Type II interferon signalling activates STAT1, which translocates to the nucleus to induce transcription of ISGs containing GAS elements in their promoters. Some proinflammatory cytokines (pro-IL-1B, pro-IL-18) are produced in an inactive form. The processing of these cytokines into their bioactive form is mediated by Capase-1, which is activated by the NLRP3 inflammasome pathway

The airway epithelium is the primary site for viral infection and replication. Viruses must first penetrate the mucus layer, which provides a first line of defence against invading pathogens. When this mechanism fails, the innate immune system is activated. Innate immunity relies on a series of germ-line encoded receptors that are expressed on epithelial and innate immune cells. These pattern recognition receptors (PRR) are sensors for pathogen-derived, evolutionarily conserved molecular structures, known as pathogen- or microbe-associated molecular patterns (PAMP/MAMP). PRR can also detect endogenous molecules called damage-associated molecular patterns (DAMP). DAMPs are normally sequestered, but released from damaged, dying or infected cells to alert the immune system to the presence of “danger”. There are four major classes of PRR; the Toll-like receptors (TLR), nucleotide-binding oligomerization domain (NOD)-like receptors (NLRs), retinoic acid-inducible gene-I (RIG-1)-like receptors (RLR), and C-type lectin receptors (CLR). Most TLRs are expressed at the cell surface (TLR1, −2, −4, −5, −6, 10), where they interact with bacterial components and viral proteins. For instance, TLR2 is a sensor for bacterial peptidoglycans, lipoproteins, RSV and RV capsid [21, 22]. TLR4 is a sensor for bacterial lipopolysaccharide and RSV F protein, and also responds to DAMPs (e.g. oxidized phospholipids, HMBG1, S100A9) during IAV infection. However, these responses are thought to be detrimental to the host as antagonizing TLR4 signalling during IAV infection can protect mice from lethal disease [23–25]. The remaining TLRs (TLR3, −7, −8, −9) are expressed in endosomes, and RLRs (e.g. RIG-I, MDA5) and NLRs are expressed in the cytoplasm. RNA sensors that elicit responses to RSV include RIG-I, TLR3, TLR7 and Nod2 (NLRC2) [26]. MDA5, TLR3, TLR7, NLRX-1 and PKR mediate responses to RV RNA [27–30]. IAV is detected by RIG-I, TLR3 and TLR7 [31]. The major viral sensing molecules are shown in Fig. 1. Host responses to infection can also be triggered by fusion between viral envelopes and the cell membrane, and virus-induced endoplasmic reticulum stress [32–34].

### Induction of proinflammatory and antiviral responses

PRR sensing of viruses triggers a series of intracellular signaling cascades that converge on two major families of transcription factors - nuclear factor kappa-light-chain-enhancer of activated B cells (NFκB) and interferon regulatory factor (IRF) (Fig. 1). These transcription factors translocate to the nucleus where they upregulate their respective proinflammatory and antiviral programs [35, 36]. The major signaling pathways involved are illustrated in Fig. 1.

The interferon system is essential for immunity to most viruses. Innate antiviral responses are primarily mediated by type I (IFN-α, IFN-β) and type III interferons (IFN-λ1, IFN-λ2, IFN-λ3), whereas type II interferon (IFN-γ) promotes macrophage activation and Th1 differentiation [37]. Interferon signaling induces the expression of hundreds of genes known collectively as interferon stimulated genes (ISG, Fig. 1). Almost 1000 genes are upregulated by type I interferon in one or more major immune cell populations, and a core set of 166 genes are upregulated across all cell types [38]. This core set includes archetypal ISGs (e.g. Mx proteins, OAS, PKR, viperin), which induce a robust antiviral state to restrict viral replication and prevent the spread of infection to neighboring cells. Mx proteins trap viral components (e.g. IAV PB2 and nucleocapsid) preventing nuclear import and viral replication. OAS activates RNaseL, which degrades viral and cellular RNA. PKR phosphorylates eukaryotic translation initiation factor eIF2α to shut down the translation of cellular and viral proteins. IFITM proteins block virus-host membrane fusion in the endosome. Although the function of most ISGs is unknown, high throughput functional screens suggest that inhibition of viral translation is a common antiviral strategy [39].

In addition to their classical antiviral function, interferons play diverse roles in infection. For example, interferon signalling drives lymphopenia [40]. Interferons also contribute to tissue injury by upregulating the expression and release of TRAIL, which induces apoptosis in airway epithelial cells [41]. Another emerging function is the promotion of virus-induced sickness behavior and cognitive dysfunction. This is mediated by upregulation of IFNAR1 on brain endothelial and epithelial cells, which in turn produce CXCL10, and this signals via neuronal CXCR3 to inhibit synaptic plasticity [42].

### Molecular logic of the innate immune system

**Fig. 2:**
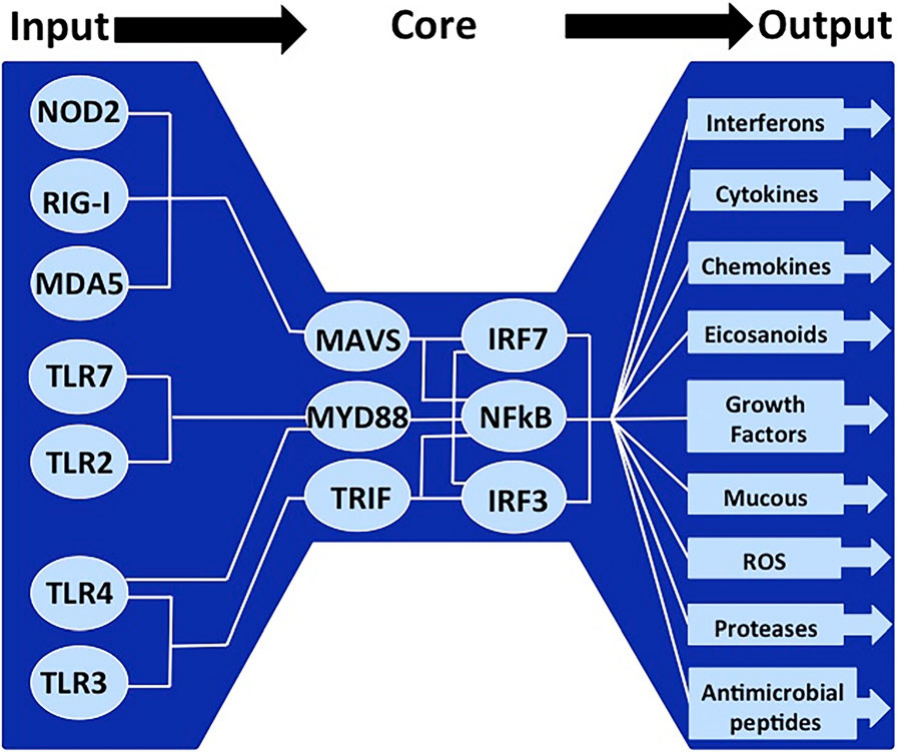
Innate immunity is governed by a bow-tie architecture. The bow-tie is a multilayered control system, comprising input, core and output signals

The molecular logic underlying innate immune responses to environmental cues is governed by a bow-tie architecture [43]. The bow-tie is an ordered control system, comprised of three crucial elements: (i) input signals or “fan-in”; (ii) the core or “knot”; and (iii) output signals or “fan-out” (Fig. 2). Complex input signals are received by the core, compressed and processed into simpler signalling pathways and converted again to complex functional output signals. Both input and output signals are highly variable, flexible, robust and diverse. If one element fails, another can take over and so the system is resistant to malfunctioning parts. In contrast, the core is specialized, efficient and rigid. This is important, because although the input and output layers are perturbed by genetic variation, which creates a lot of noise and complexity, ultimately a few core pathways are critical for activation of the innate immune response. Indeed, null mutations in core components like IRF7 can result in severe phenotypes [44]. Notably, these mutations are extremely rare, which underscores the importance of this pathway. This principle has been exploited by viruses, which target core molecules to evade the host response. For instance, RSV non-structural proteins suppress the activation and nuclear translocation of IRF3 [45], and inhibit the interaction between RIG-I and MAVS, thus abrogating the production of interferons [46]. IAV NS1 protein also inhibits IRF3 activation and interferon production [47].

### Viral challenge studies

Viral challenge studies of adult volunteers with live respiratory viruses provide a powerful model to identify host responses underlying disease pathogenesis in vivo. In these challenge models, around half of the inoculated subjects develop respiratory symptoms and have confirmed viral shedding, whereas the remaining subjects are asymptomatic [3, 48]. These studies have found that symptomatic infections are associated with a heighted immune response in blood [3, 49]. Host response signatures can therefore be leveraged to diagnose viral infections, prior to the onset of peak clinical symptoms, and can also differentiate between RSV, or RV and influenza infections [48, 49].

**Fig. 3:**
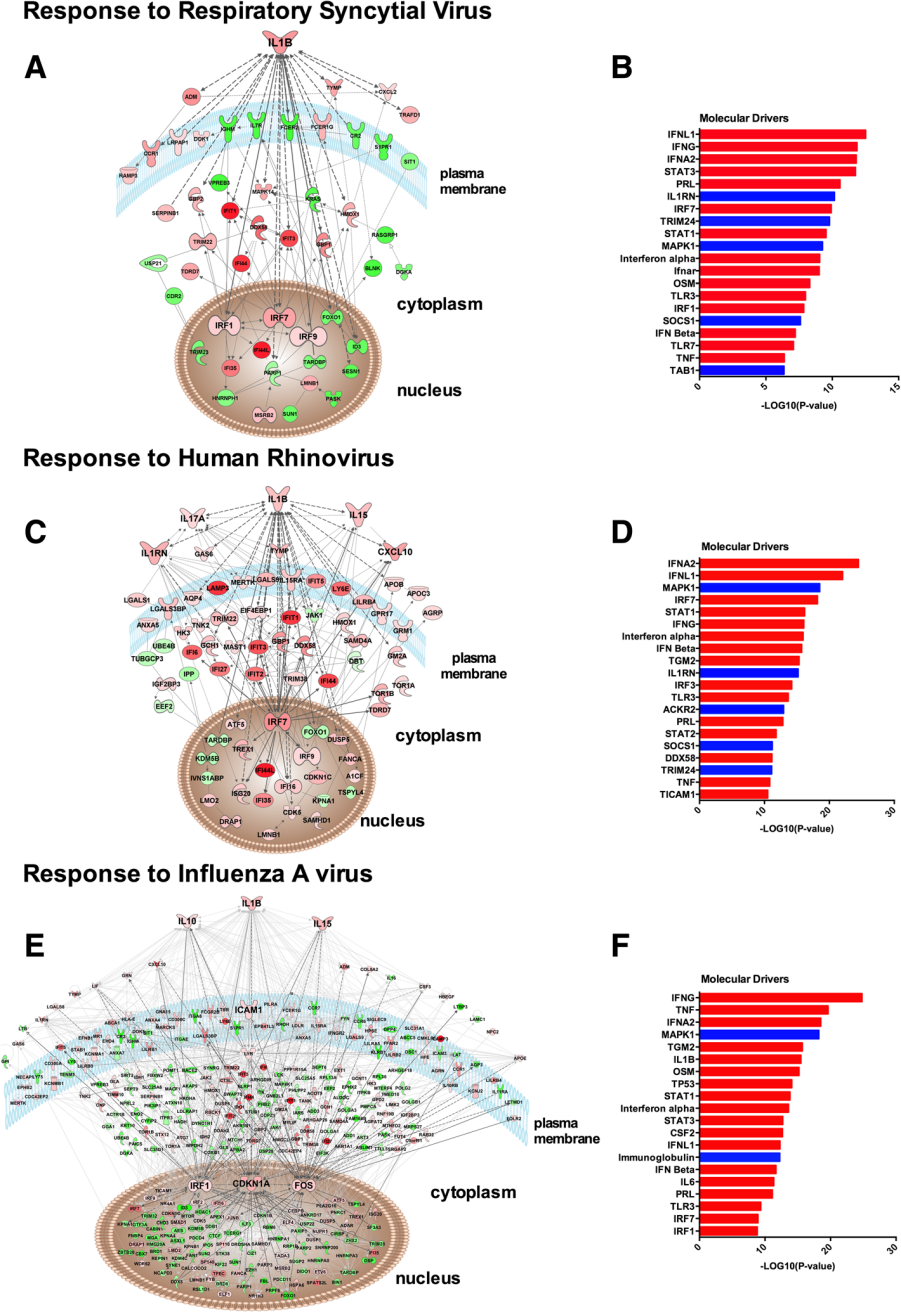
Gene networks and molecular drivers underlying symptomatic host responses to infection. Gene expression patterns were profiled in blood from adult volunteers after experimental infection with RSV, RV or IAV [3]. Differentially expressed were identified between symptomatic versus asymptomatic subjects for each virus, resulting in 934 differentially expressed genes for IAV (adjusted *p*-value < 0.05), 173 genes for RV (adj *p*-value < 0.05), and 130 genes for RSV (adj *p*-value < 0.1). **a**/**c**/**e**; Network wiring diagrams were constructed utilizing experimentally supported findings from prior studies (Ingenuity Systems KnowledgeBase) [50]. Genes coloured red were upregulated and those coloured green were downregulated. **b**/**d**/**f**; Upstream regulator analysis was employed to identify the molecular drivers of the response [51]. The negative log *p*-value was shaded red to indicate pathway activation, and blue indicates inhibition

To characterize the molecular networks underlying symptomatic infections, we downloaded a microarray data set from a viral challenge study in which adult volunteers were inoculated with RSV, RV or IAV [3]. We employed case/control comparisons of symptomatic versus asymptomatic subjects to identify differentially expressed genes for each virus, and we found that the strongest disturbance of the transcriptome was induced by IAV, followed by RV and then RSV. To provide a network-level view of these data, we leveraged experimentally supported molecular interaction data from prior studies to reconstruct the wiring diagram of the underlying gene networks [50]. These networks unveil the innate immune response hubs underlying symptomatic infections (Fig. 3a, c and e). To identify the putative causal pathways that give rise to the observed gene expression patterns, we employing upstream regulator analysis [51]. This analysis suggested that symptomatic responses to RSV were largely driven by IFN-λ, IFN-γ, IFN-α and STAT3 signalling (Fig. 3b). RV responses were also characterized by upregulation of type I, II and III interferon signalling, but lacked a prominent STAT3 signature (Fig. 3d). The hallmark of the IAV response was upregulation of IFN-γ, TNF, IFN-α and IL-1β signalling (Fig. 3f). The proinflammatory component of the IAV response provides a plausible mechanism to explain why this virus elicits a stronger perturbation to the transcriptome.

### Pathogenesis of RSV infections

Most children with RSV develop mild symptoms such as cough, fever, sore throat and runny nose. Up to 40% of infants develop lower airway symptoms (e.g. shortness of breath, wheeze, bronchiolitis) and 0.5-2% of all infected infants are hospitalized [52]. Risk factors for severe disease include premature birth, low birth weight, young age, male gender, chronic lung disease, congenital heart disease, immune deficiency, low socio-economic status and tobacco exposure [52]. The presence of pathogenic bacteria (*Streptococcus*, *Moraxella*, *Haemophilus*) in the nasopharyngeal microbiome increases the risk of the infection spreading from the upper to the lower airways and inducing more severe symptoms [53].

The pathogenesis of RSV infections is thought to be mediated by direct cytopathic effects of the virus on lung epithelial cells in combination with the inflammatory response [54–56]. Airways obstruction results from epithelial damage, including loss of function of motile cilia, epithelial sloughing, apoptosis, mucous hypersecretion, edema of the airway wall, and infiltration of neutrophils and lymphocytes [2]. High viral load correlates with increased disease severity in hospitalized infants, while faster rates of RSV clearance are associated with more rapid disease resolution and shorter hospitalization [57, 58]. In differentiated epithelial cell cultures, RSV induces a relatively mild disturbance to the transcriptome, which is an order of magnitude lower than that of IAV [59]. The RSV response in this system is mainly restricted to an interferon signature, whereas the IAV response comprises type I and III interferons, and a broad range of proinflammatory cytokines and chemokines. However, these models cannot provide insight into the role of innate signalling from inflammatory cells that are recruited to the airways.

A unique and perplexing feature of RSV infections is the disproportionate impact on infants. At birth, the immune and respiratory systems are functionally immature, and susceptibility to lower respiratory illnesses can be explained in part by diminished lung function [60, 61]. On the other hand, the neonatal innate immune system has a reduced capacity to produce cytokines (IL-12, IFN-α, TNF) that promote Th1 responses against intracellular pathogens, but has a similar or augmented capacity to produce cytokines (IL-1b, IL-6, IL-8, IL-10, IL-23) that promote Th2 and Th17 responses [62, 63]. This defect in Th1 function is also apparent within the CD4 T cell compartment [60], and is thought to result in Th2- and Th17-skewed responses to infection [64–66]. Recent evidence from parallel studies in neonatal animal models and in hospitalized infants suggests that RSV-induced IL-33 responses are exaggerated in early life, which in turn activates group 2 innate lymphoid cells (ILC2) that drive Th2-associated pathology (eosinophilic inflammation, mucus hypersecretion, airway hyperresponsiveness) [67]. Th1/Th2 imbalance during RSV bronchiolitis is modified by interactions between TLR4 genotype and environmental LPS exposure levels, thus explaining in part why some individuals are predisposed to severe disease [68].

Gene network patterns in blood have been investigated in a cohort of hospitalized infants with RSV, RV or IAV [69]. RSV response networks were characterized by upregulation of interferon signalling, innate immunity and neutrophil/myeloid signatures, and downregulation of lymphocyte signatures (T cells, B cells, NK cells). Notably, interferon responses continued to rise at one month after hospitalization, whereas expression of the other networks faded. Compared with RV or IAV, RSV was associated with overexpression of neutrophil genes and marked suppression of lymphocyte genes. Younger infants (<6 months) with RSV had impaired innate immune and inflammatory responses compared with older infants. The severity of RSV illness was correlated with heightened expression of networks related to neutrophils, inflammation and erythrocytes, whereas innate immune and interferon responses were similar in moderate and severe infants. Finally, length of hospitalization and total duration of supplemental oxygen was strongly correlated with the extent at which the transcriptome was perturbed in infants with RSV illness relative to healthy baseline controls. These findings implicate excessive activation of neutrophil and inflammatory responses in the pathogenesis of severe RSV disease.

### Pathogenesis of RV infection

RV is the most important trigger of asthma exacerbations, however, it is frequently detected in children with asthma in the absence of significant symptoms [4]. This suggests that specific environmental conditions are necessary for RV to provoke exacerbations [70]. Natural allergen exposure in sensitized subjects markedly increases risk for experiencing a severe exacerbation with RV [71]. Pathogenic bacteria can also increase the severity of RV infections [72]. Another contributing factor is the virus itself. RV-C is the most common virus found in children presenting to hospital with acute asthma [73]. RV-C-induced exacerbations are more severe, and associated with an increased risk of recurrence, especially in atopics [73, 74]. A variant in CDHR3 (Cys_529_ → Tyr, rs6967330) that enhances RV-C binding and replication by 10-fold is associated with recurrent severe exacerbations [19, 75]. RV-B species on the other hand have reduced replication rates, elicit lower levels of cytokine production, and induce less severe respiratory symptoms compared with RV-A and RV-C [76, 77]. Antibody responses to RV-C are much lower than RV-A or RV-B, providing further evidence that RV species elicit differential immune responses [78].

There is evidence suggesting that genetic variation modulates host responses to RV and the expression of respiratory symptoms [79, 80]. Variants in the 17q21 locus are associated with RV-induced wheezing but not RSV-induced wheezing [80]. Although the underlying mechanisms are unknown, expression levels of only two genes (ORMDL3, GSDMB) in this region are induced by RV and are correlated with the 17q variants [80]. Transgenic overexpression of human ORMDL3 in mice results in increased levels of airways remodelling, a hallmark of asthma [81]. Of note, a unique aspect of the airway epithelial cell response to RV is the induction of a broad range of genes involved in epithelial repair and remodelling [82, 83]. In vitro studies have shown that the capacity of the airway epithelium to repair is impaired in subjects with asthma, and this capacity diminishes even further in the presence of RV [84]. These data highlight a potential role for dysregulated repair and remodelling in the pathogenesis of RV infections.

Numerous studies have investigated the role of innate immunity in asthma. Ex vivo studies in airway epithelial cells found that RV-induced IFN-β and IFN-λ responses were impaired in subjects with asthma, leading to increased viral replication and shedding and exaggerated secondary responses [85, 86]. However, this finding was replicated by some groups but not others (reviewed [87]). A simple explanation for these inconsistent findings is that asthma is a highly heterogeneous disease that can be divided into Th2^High^ versus Th2^Low^ subgroups [88]. Given that type I and III interferon and Th2 responses are mutually antagonistic, variations in airway epithelial cell responses to RV ex vivo may reflect the nature of the inflammatory microenvironment that was present during specimen collection rather than a defect in the capacity to respond to viruses [89–93]. Indeed, genomic studies in peripheral blood mononuclear cells (PBMC) have found that asthma exacerbations are heterogeneous conditions and comprise at least three molecular subphenotypes [94]. The first phenotype was characterized by activation of innate immunity genes. The second was enriched with antigen driven pathways of adaptive immunity and the third was not associated with any dominant biological pathways. An alternative explanation for impaired interferon responses in subjects with asthma is decreased expression of pathways that promote these responses (e.g. TLR7) or increased expression of pathways that inhibit these responses (e.g. SOCS1) [29, 95].

We have characterized the inflammatory mechanisms that underpin virus-induced asthma exacerbations in vivo. We found that interferon gene networks were upregulated in the upper (nasal wash) and lower (sputum) airways of children during acute asthma exacerbations compared to 7–14 days later [50, 96]. However, expression of Th1 and type I interferon gene networks was reduced in those asthmatic children with evidence of chronic airflow limitation, suggesting a protective role for interferons [96]. Other groups have reported that expression of interferons is increased and correlated with expression of respiratory symptoms during naturally acquired RV-induced exacerbations [97, 98]. Taken together, these studies highlight the potential for interferons to play a dual role in asthma, in both the regulation of antiviral immunity and expression of respiratory symptoms [99]. Variable expression of interferon production in asthma may also be related to viral susceptibility or loads, or variations in the timing from infection onset to peak symptom expression.

Genomic studies from our group found that childhood asthma exacerbation responses in PBMC were associated with upregulation of innate immune and Th2-associated signatures, suggesting that these two pathways interact to drive disease pathogenesis [100]. The mechanisms that link innate antiviral responses with Th2-associated effector mechanisms are not well understood, but recent studies suggest a role for IL-25 and IL-33. Expression of both of these factors is increased in the airways of asthmatic subjects after experimental RV infection in vivo [101, 102]. In allergic mice, blockade of IL-25 signalling markedly suppressed RV-induced production of mucus and airways inflammation [101]. Moreover, supernatants from RV-infected bronchial epithelial cells stimulate Th2 cytokine production in Th2 and ILC2 cells in an IL-33 dependant manner [102]. The mechanisms that regulate the production of IL-25 and IL-33 during RV infections are not well understood. Employing siRNA-mediated gene silencing, we found that IRF7 promotes innate antiviral responses to RV in airway epithelial cells, and limits IL-33 responses [103]. These data are consistent with the mutually antagonistic relationship between the interferon and Th2 response system described above.

### Pathogenesis of IAV infections

IAV is ranked amongst the top 10 causes of death in the USA [7]. Mortality rates can be even higher during pandemics such as the 1918 “Spanish Flu”, which resulted in an estimated 50 million deaths [104]. Symptoms of influenza include fever, headache, sore throat, sneezing, nausea, body aches and fatigue. Risk factors for severe disease include old age, very young age, pregnancy, immune deficiency and chronic diseases like asthma, COPD and obesity [105]. The pathogenesis of IAV infections is thought to be determined by cell tropism for human airway and alveolar epithelial cells, viral replication rate and the intensity and dynamics of the inflammatory response [105]. Large swathes of epithelium are denuded or damaged by IAV infection, resulting in exudation of fluid into the airways and alveolar spaces, and in severe cases this can lead to hypoxaemia and respiratory failure [105]. A robust repair response is crucial to restore the epithelial barrier and lung microarchitecture following viral clearance [106]. However, the mechanisms that act to resolve inflammation and restore tissue homeostasis also impair immunity, and this increases susceptibility to secondary bacterial pneumonia, especially during pandemics [107].

Host responses to IAV infection in the lung are highly dynamic and comprise multiple sequential waves of gene expression, and similar findings have been reported in blood [108, 109]. The first wave peaks around 2–5 days post infection, and is characterized by upregulation of innate immune networks, interferon and NK cell responses, and proinflammatory cytokines and chemokines. The next wave spikes on day 8 post infection, and this is defined by upregulation of T cell activation and induction of apoptosis. This is followed by upregulation of B cell activation and proliferation, which is maximal around day 14. On day 30, differentiation and tissue repair processes are upregulated, and these responses persist out to 60 days post infection, suggesting long term or permanent alterations to the lung [108]. Notably, restoration of epithelial barrier integrity and lung function after IAV infection depends on an IL-33-ILC2-amphiregulin axis [110]. This contrasts with the known pathogenic role of IL-33/ILC2 in the context of RSV and RV illness.

The biology of asymptomatic IAV infections is poorly understood. Viral challenge studies are particularly informative in this context, because only a subset of the participants develops symptoms and has evidence of viral shedding [3, 111]. Of the remaining asymptomatic subjects, a subset of them has detectable viral shedding, albeit levels are lower and delayed in onset [111]. The temporal dynamics of the host response to IAV in blood is strikingly different in symptomatic versus asymptomatic subjects [111]. Expression of clinical symptoms is strongly associated with upregulation of viral sensing pathways and interferon-stimulated genes, as well as neutrophil activation, proinflammatory responses and inflammasome signaling (NOD2, NALP3, CASP5, IL-1b) [111]. In contrast, a hallmark of the asymptomatic response was upregulation of pathways that restrain cytokine signaling (SOCS2, SOCS5) and oxidative stress (SOD1, STK25), together with suppression of inflammasome signaling pathways. Of note, myeloid derived suppressor cells (MDSC) accumulate in the lung during IAV infections, and may contribute to the immunosuppressive environment via production of IL-10 [112, 113]. These findings suggest that a unique host response is mobilized during asymptomatic infections, and this could potentially be leveraged to develop novel therapeutics.

Genomic studies of host responses to highly pathogenic avian H5N1 virus and the 1918 (H1N1) pandemic virus have provided unique insights into disease pathogenesis. The 1918 virus was recreated from the genomic sequence using reverse genetics [114]. In mice and cynomolgus macaques, 1918 infection results in a lethal disease phenotype, which is characterized by high viral titers, severe lung pathology and marked and sustained activation of interferon-stimulated genes and pro-inflammatory cytokines and chemokines until death [115, 116]. Notably, expression of multiple type I interferon subtypes was suppressed despite high levels of ISGs, suggesting that the 1918 virus elicits an aberrant interferon response, and/or has reduced sensitivity to the interferon system. Highly pathogenic avian H5N1 infections are also characterized by severe lung pathology together with early and sustained activation of proinflammatory and interferon responses [117, 118]. One interpretation of these data is that although a robust innate immune response becomes activated, it is not able to control the virus, resulting in death due to direct viral damage to the airways. An alternative view is that an unconstrained “cytokine storm” drives lethal disease [119]. With regards to the former hypothesis, the virulence of the 1918 viral proteins has been demonstrated by introducing them into less pathogenic strains. These experiments found that the 1918 HA protein confers high viral loads and aberrant host responses resulting in mortality, whereas the polymerase and NP proteins confers high viral loads, but does not induce lethal disease [120]. The NS1 protein blocks interferon signaling and lipid metabolism, the PB2 protein enhances inflammation and suppresses pathways involved in lung repair [121, 122] and a mutation in the 1918 NP protein confers resistance to Mx1 [123]. These data shed light on the exceptional virulence of the 1918 virus. With regards to the cytokine storm hypothesis, attempts to curtail the 1918-induced host response using gene deficient mice can prolong (Nos2−/−, TNFR−/−) or shorten (IL1R1−/−, IFNAR1−/−) survival, but does not prevent mortality [118, 124, 125]. In contrast, therapy with the ROS scavenger EUK-207 increased survival in mice with 1918 [126].

It is important to acknowledge that the findings from gene deficient mice are oversimplified, because immune mechanisms that are important in viral control may also damage the host, and this dual role of the immune system may be dependent on the timing and magnitude of gene expression. In this context it is noteworthy that lethal influenza in mice is associated with excessive activation of neutrophils. Increased survival in this model was achieved by attenuating rather than ablating the neutrophil response, without compromising viral clearance [127].

## Conclusions

The pathogenesis of respiratory viral infections involves the complex interplay between viral virulence factors, environmental conditions, the magnitude and temporal dynamics of the host response, and host susceptibility factors. Symptomatic infections are associated with increased viral shedding and a heightened host immune response. The disproportionate impact of respiratory viral infections in early life and in individuals with chronic respiratory diseases like asthma can be explained by an aberrant host response. It is also apparent that the immune response whilst essential for viral control also promotes the expression of respiratory symptoms and causes collateral damage to the tissues, which in some cases can lead to mortality. This dual role of the immune system can be difficult to dissect using conventional knockout mouse models, thus more systems-levels analyses of multi-omic data sets will be essential to unlock the underlying mechanisms.
